# Blue as an Underrated Alternative to Green: Photoplethysmographic Heartbeat Intervals Estimation under Two Temperature Conditions †

**DOI:** 10.3390/s21124241

**Published:** 2021-06-21

**Authors:** Evgeniia Shchelkanova, Liia Shchapova, Alexander Shchelkanov, Tomohiro Shibata

**Affiliations:** 1 Graduate School of Life Science and Systems Engineering, Kyushu Institute of Technology, 2-4 Hibikino, Wakamatsu, Kitakyushu, Fukuoka 808-0135, Japan; tom@brain.kyutech.ac.jp; 2 Omsk State Technical University, 11 Prospekt Mira, 644050 Omsk, Russia; li.shchapova@mail.ru (L.S.); alshche@gmail.com (A.S.)

**Keywords:** photoplethysmography, blue light, color sensor, heartbeat intervals

## Abstract

Since photoplethysmography (PPG) sensors are usually placed on open skin areas, temperature interference can be an issue. Currently, green light is the most widely used in the reflectance PPG for its relatively low artifact susceptibility. However, it has been known that hemoglobin absorption peaks at the blue part of the spectrum. Despite this fact, blue light has received little attention in the PPG field. Blue wavelengths are commonly used in phototherapy. Combining blue light-based treatments with simultaneous blue PPG acquisition could be potentially used in patients monitoring and studying the biological effects of light. Previous studies examining the PPG in blue light compared to other wavelengths employed photodetectors with inherently lower sensitivity to blue, thereby biasing the results. The present study assessed the accuracy of heartbeat intervals (HBIs) estimation from blue and green PPG signals, acquired under baseline and cold temperature conditions. Our PPG system is based on TCS3472 Color Sensor with equal sensitivity to both parts of the light spectrum to ensure unbiased comparison. The accuracy of the HBIs estimates, calculated with five characteristic points (PPG systolic peak, maximum of the first PPG derivative, maximum of the second PPG derivative, minimum of the second PPG derivative, and intersecting tangents) on both PPG signal types, was evaluated based on the electrocardiographic values. The statistical analyses demonstrated that in all cases, the HBIs estimation accuracy of blue PPG was nearly equivalent to the G PPG irrespective of the characteristic point and measurement condition. Therefore, blue PPG can be used for cardiovascular parameter acquisition. This paper is an extension of work originally presented at the 42nd Annual International Conference of the IEEE Engineering in Medicine and Biology Society.

## 1. Introduction

Green is the most widely used wavelength in reflectance PPG. It is considered to provide the most acceptable signal quality and artifact resistance. Meanwhile, deoxy-hemoglobin and oxy-hemoglobin show the highest absorption in the blue part of the spectrum, at 420 μm and 410 μm, respectively [1]. Nonetheless, in the field of PPG, blue wavelengths have been often overlooked because they are assumed to have an insufficient penetration depth. We found only two published studies comparing the PPG in blue light to other wavelengths [2,3]. Both of them examined the susceptibility of red, green, and blue PPG signals to motion artifacts. Although blue and green light PPGs showed nearly equivalent artifact resistance, Bland-Altman analysis demonstrated the green PPG’s superiority in heart rate estimation. It is worth mentioning that these studies used a biased approach to the wavelengths’ comparison since the photodetectors employed had inherently lower sensitivity to blue compared to green wavelengths: Lee et al. used TEMD5510FX01 (Vishay semiconductors) detector with peak sensitivity at 540 nm; and Matsumura et al. the Bayer pattern video camera with the green channel being half of the entire image sensor.

In real-life scenarios, temperature-related artifacts are as crucial as motion-induced signal corruption. However, to the best of our knowledge, the temperature-related changes have not been explored sufficiently. Maeda et al. [4] proved the advantage of green PPG over infrared (IR) PPG at two skin temperatures in terms of the AC/DC ratio and the accuracy of heart rate (HR) detection for these signals. Our previous study was the first one investigating blue (B) PPG in comparison with green (G) PPG in terms of the amplitude and accuracy of HBIs estimation under two temperature conditions [5]. As a photodetector, we employed a TCS3472 Color Sensor (ams AG), which is equally sensitive to blue, green, and red parts of the light spectrum. The preliminary results showed that the B PPG performance was comparable to G PPG under both temperature conditions.

Similar to studies [2,3,4], in our pilot study, photoplethysmographic HBIs measurements were based on systolic peak detection. This characteristic point has been extensively used in studies on heart rate variability (HRV) and pulse arrival time estimation for its detection algorithm’s simplicity. However, a growing body of research has indicated that the peak (PPGmax) might not be the most optimal choice in HBI estimation and put forward other characteristic points on the PPG wave [6,7]. In experimental studies on HBIs and HRV measurements in resting individuals [7,8,9,10], maximum of the first PPG derivative (VPGmax), maximum of the second PPG derivative (APGmax), minimum of the second PPG derivative (APGmin), and intersecting tangents (IT) are conventionally used PPG points, which were suggested as a better alternative to PPGmax.

Due to the promising results obtained in our preliminary study, B PPG under two temperature conditions was examined in a larger number of subjects in the present study. In order to verify the validity of the results in more detail, the HBIs of the B and G PPG waveforms at each temperature condition were measured at five characteristic points, namely, PPGmax, VPGmax, APGmax, APGmin, and IT. The comparative evaluation of B and G PPGs show that the accuracy of the HBI estimation of B PPGs is comparable to that of G PPGs, regardless of the measurement points and conditions.

Blue light is widely used in therapeutic interventions, such as in newborn jaundice [11], acne vulgaris [12], and photodynamic therapy for pre-cancerous skin lesions [13]. Exploring blue PPG could open up the possibility of incorporating cardiovascular monitoring functionality into light-based therapeutic devices.

The structure of this paper is as follows. Section 2 introduces the signal acquisition setup. Section 3 introduces the experimental results. Section 4 is devoted to the discussion. Section 5 presents the conclusion.

## 2. Materials and Methods

**Figure 1:**
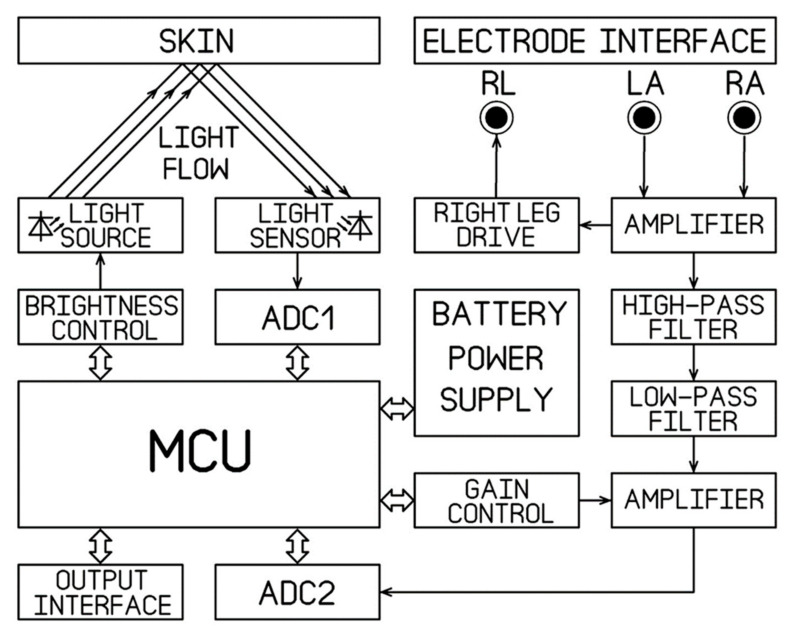
The system block diagram.

**Figure 2:**
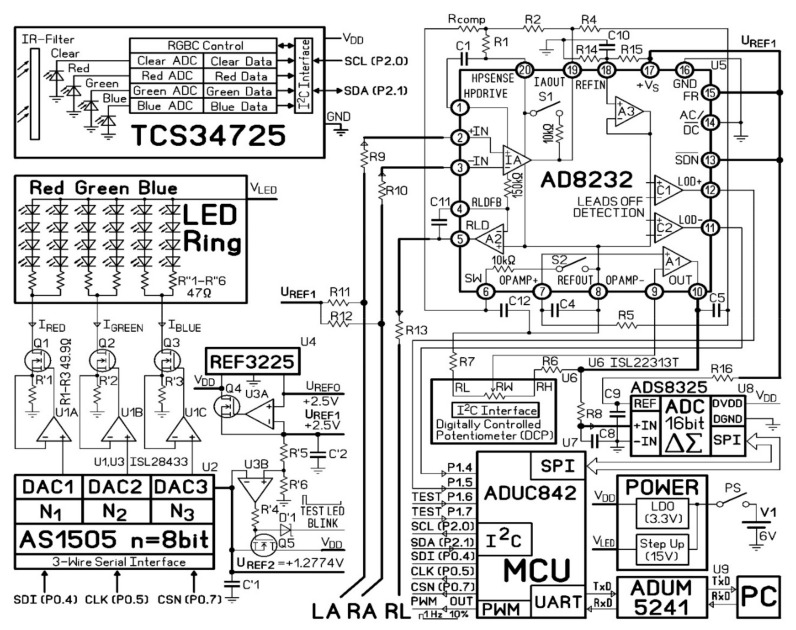
The circuit diagram.

For our application, we designed a PCB board based on the ADuC842 micro converter (Analog Devices Inc., Wilmington, MA, USA), which features low inherent noise and low power consumption. One rechargeable battery 3.5–8.4 V or 5 V adapter provides the system power supply. Figure 1 illustrates the system block diagram. The circuits of the TCS3472 sensor, the illumination unit, ECG unit, ADuC842 micro converter, and power supply are presented in Figure 2.

### 2.1. The PPG Measurement System

The B and G PPG signals were acquired with the custom-made PPG system with TCS3472 Color Sensor (ams AG) as a photodetector. The illumination and signal detection were performed normally to the skin surface.

**Figure 3:**
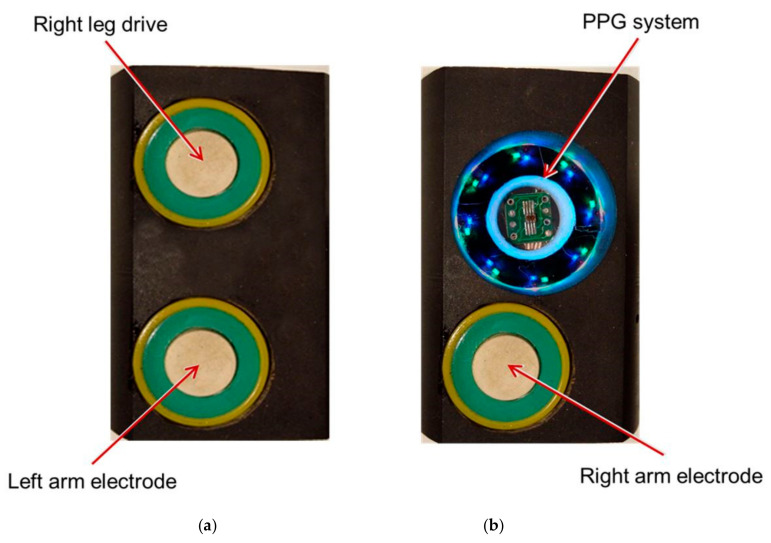
The signal acquisition modules for the (**a**) left hand (Left Arm and RLD electrodes) and (**b**) right hand (Right Arm electrode and PPG device).

The light source was designed with Inolux IN-S63BT series LEDs with wavelength peaks 530 nm and 630 nm. There were eight LEDs of each type, mounted on a circular circuit board with the inner and outer diameters of 23 mm and 30 mm, respectively. The LED illumination ring was inserted into a cylindrical screening spacer (13 mm between the opening and the LED ring) to prevent ambient light interference. For PPG signal acquisition, the hand is placed on the spacer’s rim so that a round spot of palm skin diameter 30 mm is illuminated from the bottom by the LED ring. The TCS34725 sensor was mounted in the center of the LED ring. (Figure 3b) The sensor captures the skin-remitted light intensity over a two-dimensional grid, consisting of an equal number of photodiodes for red, green, blue, and clear light sensing. Its ADCs simultaneously output averaged values separately for each of the four channels. The integration time of 4.8 ms (two steps) was chosen as a tradeoff between the light intensity resolution (ADC max count = 2^11^) and the sampling rate (100 Hz). The gain was set at × 60.

The programmable LED luminance controller system varies the light source brightness. LED configuration is a combination of parallel and series. Three voltage output digital-to-analog converters (DACs) provide the programmable voltages for the respective LED groups (red, green, and blue). A current mirror (operational amplifier U1, resistor R’, field-effect transistor Q) operates as a voltage-controlled current source and can provide a constant current ranging 0–25.5 mA per LED group with 0.1 mA resolution.

The current fed to each LED group (Ired, Igreen, Iblue) is defined by the formula:

(1) $$I_{LED\;i}=\frac{U_{REF2}\times N_{i}}{R_{i}^{’}\times 2^{n}}$$

where *U_REF2_* is the DAC reference voltage, *N_i_* is the binary input to the respective DAC, *R^’^_i_* is the resistor value, and *n* is the DAC resolution.

Given the difference in skin optical properties among subjects, the LED illumination intensity is adjusted automatically to prevent the TCS3472 readings from going off its analog-to-digital converters (ADCs) scale. This calibration is performed at the beginning of each measurement. After the system is turned on, the current 25.5 mA is fed to each LED group. After obtaining the initial green and blue readings, the current is adjusted until the largest amplitude signal (either green or blue) reaches 63% of the respective ADC scale. The current fed to the other LED group is adjusted proportionally by an iterated successive approximation algorithm.

### 2.2. Electrocardiography (ECG) Measurement System

The ECG was custom-made with the AD8232 front end (Analog Devices Inc., Wilmington, MA, USA). The AD8232 features an instrumentation amplifier, a reference buffer, a spare amplifier, an amplifier to inject back the common-mode voltage, and several digital features such as shutdown mode, AC, or DC leads-off detection, and Fast Restore.

The first stage is a high-impedance instrumentation amplifier that amplifies the small biopotential signal by a factor of 100. Together with R_1_, R_2_, C_1_, and C_2_, it provides a two-pole high-pass filter set at 0.48 Hz. Therefore, this first stage is a high pass filter and makes the AD8232 front end an AC coupled system. The first stage gain is 40 dB. The second stage is formed by a spare amplifier configured (with R_4_, R_5_, C_4_, and C_5_) as a second-order 37 Hz Low Pass Filter with additional gain. The second stage gain can be changed according to the formula:

(2) $$GAIN_{2}=\frac{R_{6}}{R_{7}}+1\;$$

**Figure 4:**
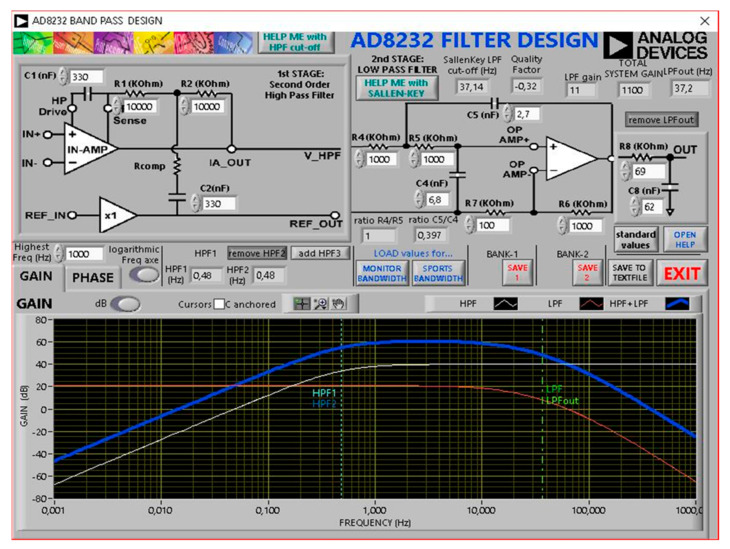
The frequency response plot.

U3C buffers the input of ADC U8. The additional low-pass filter (R_8_C_8_) has cut-off frequency 37 Hz. The frequency response of the ECG system was calculated using “AD8232 Filter Design Tool” software (Analog Devices Inc., Wilmington, MA, USA) (Figure 4).

The main features of the ECG acquisition block: 1 Channel; 500 samples/s; 115 kBaud/s; Right leg drive (RLD) circuit. The ADuM5241 digital isolator (Analog Devices Inc., Wilmington, MA, USA) is employed for human safety and noise protection. ADS8325 16-bit ADC converts the analog output of the AD8232 to a digital signal.

Standard Lead I ECG signals were recorded with the three-electrode configuration. Although gel Ag/AgCl electrodes are considered the gold-standard electrodes in clinical measurements, they were not practical for signal acquisition in our experimental setup. Instead, we opted for nanoelectrodes developed in the Laboratory of Medical Engineering, Tomsk Polytechnic University. They exhibit low noise and electrode-skin impedance and allow biosignal recording with both wet and dry contact without any contact-enhancing substances [14]. The material of the electrodes is highly durable, with no deterioration of characteristics over time. All three electrodes are around 1 cm in diameter.

The PPG system and the Right Arm ECG electrode were inserted into a plastic holder. This module was used for the signals acquisition from the right palm. The other two electrodes (Left Arm and RLD) were installed on a similar plastic holder and comprised a sensing module for the left hand. (Figure 3)

### 2.3. Participants and Experimental Procedure

Twelve healthy volunteers, ten men and two women between 24 and 59 years old, participated in the study. The experiments were conducted based on the principles of the Helsinki declaration. The experiments were carried out under room temperature 24 ± 2 °C. During the acquisition process, the participants were asked to sit still and breathe normally with both hands resting on the table at a height equivalent to their heart position. The left palm was placed on the sensor module with RLD and Left Arm ECG electrodes, while the right palm was placed on the module with the Right Arm electrode and PPG unit.

The data were collected under two protocol conditions. Under the baseline condition, the subjects kept the hands in warm (40 °C) water until the local skin temperature reached 34 + 2 °C. Then, the signals were acquired after the Cold test: the skin temperature was lowered to 20 + 4 °C by immersion in ice water. The temperature was measured using an optical pyrometer. For each subject, the G and B outputs of TCS3472 were recorded simultaneously for 2 min along with the ECG signal.

### 2.4. Signals Acquisition and Processing

**Figure 5:**
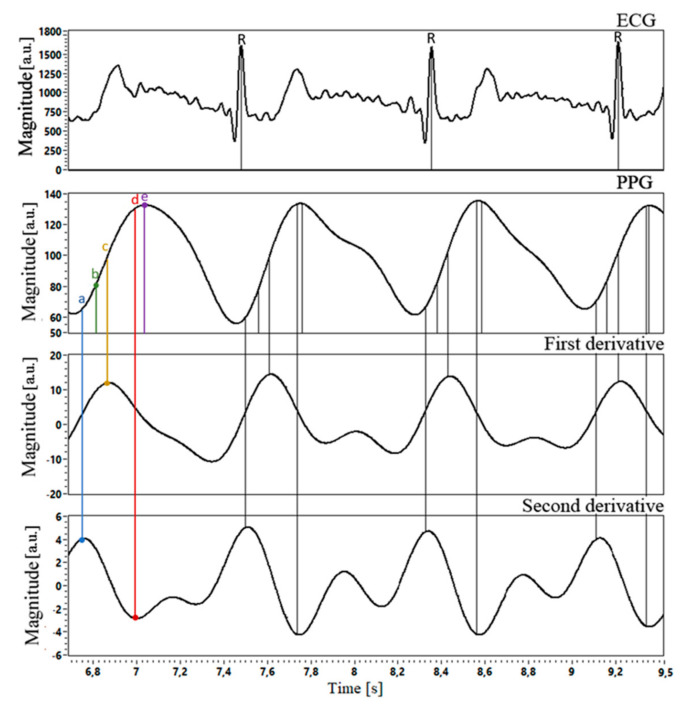
Example of the ECG and respective PPG waveforms with 1st and 2nd PPG derivatives. The characteristic points (a-e) are indicated on the PPG waveform: (**a**) intersecting tangents (IT) (**b**) maximum 2nd derivative (APGmax) (**c**) maximum 1st derivative (VPGmax) (**d**) minimum 2nd derivative (APGmin) (**e**) PPG maximum value (PPGmax).

Data acquisition and processing were performed with the original software, created in LabVIEW18.0.4 (National Instruments, Austin, TX, USA). The instantaneous ECG values, as well as the B and G PPG readings, were captured simultaneously. The ECG signals were passed through a fourth-order Chebyshev I filter with 1 Hz to 12 Hz cutoff frequencies. For the PPG signals, we used a bidirectional fourth-order Chebyshev II filter with a 0.5–6 Hz passband range. False-positive beats were removed by moving average and subsequent visual inspection. From each recording, we chose 60 HBIs for further analysis. Heartbeat intervals (HBI) were measured using R-wave on the ECG signals (R–R interval) and five characteristic points on the PPG signals (PPGmax-to-PPGmax, VPGmax-to-VPGmax, APGmax-to-APGmax, APGmin-to-APGmin, IT-to-IT). Characteristic points detection on ECG and PPG waveforms is shown in Figure 5.

### 2.5. Signals Alignment

**Figure 6:**
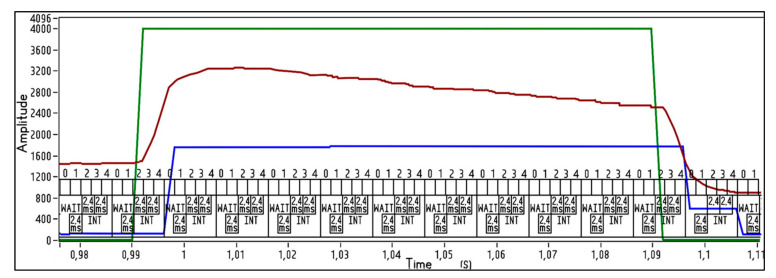
Alignment of the ECG and PPG Clear signals with the test impulse. Digits from 0 to 4 shows the numbers of times the ECG signal is sampled in 10 ms.

To ensure that the beat intervals on the PPG waves correspond to the same heartbeats on ECG, we created a test square impulse (TTL, 2.5 V, 10% duty cycle, 1 Hz). Via frequency compensated voltage divider 1:2500, it was fed from PWM out to AD8232 inputs and P1.7 port of ADuC842 to put the LED ring into blink mode. This test square signal was inserted in each data packet transmitted to the PC. As seen in Figure 6, PPG lags behind ECG by about 4.8 ms.

### 2.6. Statistical Analyses

The data (the ECG and PPG-measured HBIs values and the differences between them) were tested the normality using the D’Agostino-Pearson test and were found to be non-normally distributed. The results were recognized as significant if the computed *p*-value was < 0.05.

Bland-Altman analysis was performed for B and G PPG evaluation with respect to the ECG. It is a standard method in validation studies, where a novel method is tested against a well-established one. The analysis results are represented by a scatter plot, where the Y-axis shows the difference between the paired measurements and the X-axis represents their average value. In this study, since the ECG is considered the ground truth for HBIs calculation, the mean differences between the ECG and PPG are plotted against the ECG values. The data from 12 subjects were pooled, yielding 720 ECG-PPG data pairs for each characteristic point in G and B PPG waveforms. Since in the Bland-Altman method, the calculation of bias and limits of agreement is based on the normality assumption [15], we firstly log-transformed the ECG and PPG time series to approximate normal distribution.

The bias was calculated by averaging the differences between the values measured by two methods. The limits of agreement (LoA) were calculated using the standard deviation of the differences. The 95% LoA assume that 95% of the differences between the two methods lie within this interval. The bias and LoA specify the accuracy and precision, respectively. The bias is considered significant if the line of equality is not within the 95% CI of the mean difference. In addition to bias and LoA with their respective confidence intervals (CI), Bland-Altman ratio (BAR) and acceptable limit of agreement (ALoA) were calculated, as these evaluation criteria are commonly used in studies on PPG validation. We also calculated the bias, normalized to the average ECG value (% bias), to eliminate its dependency on HBIs durations. BAR was defined as the ratio of half the agreement limits to the mean of (ECG + PPG) averaged values. Conventionally, agreements are classified as good (BAR < 0.1), moderate (0.1 ≤ BAR < 0.2), or poor (BAR ≥ 0.2). The ALoA was defined as 20% of the mean of (ECG + PPG) averaged values.

## 3. Results

### 3.1. Bland-Altman Analysis

In all B and G PPG data under investigation, the bias was low compared to the ECG intervals duration (% bias ranged from 0.2% to 0.9%); the 95% CI of bias contained the value zero, implying the absence of a significant systematic error. Notably, the independent sample t-test revealed no significant difference in the bias values between the two types of PPG signals under both conditions for all characteristic points used in the analysis (the results were recognized as significant at *p* < 0.05.). Furthermore, both B and G PPG demonstrated BAR < 0.1 and LOA less than ALoA. To sum up, Bland-Altman analysis suggests good consistency of HBIs, obtained by the G PPG and B PPG, with the ECG measured values under both protocol conditions.

**Figure A1:**
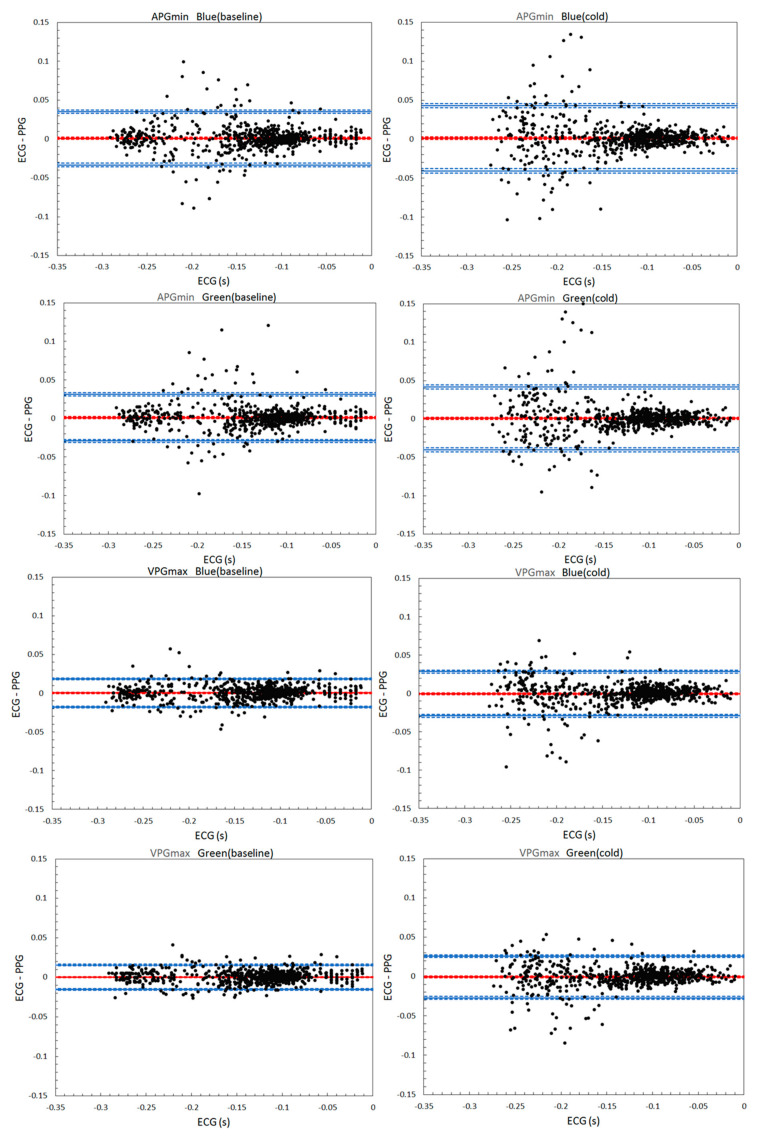

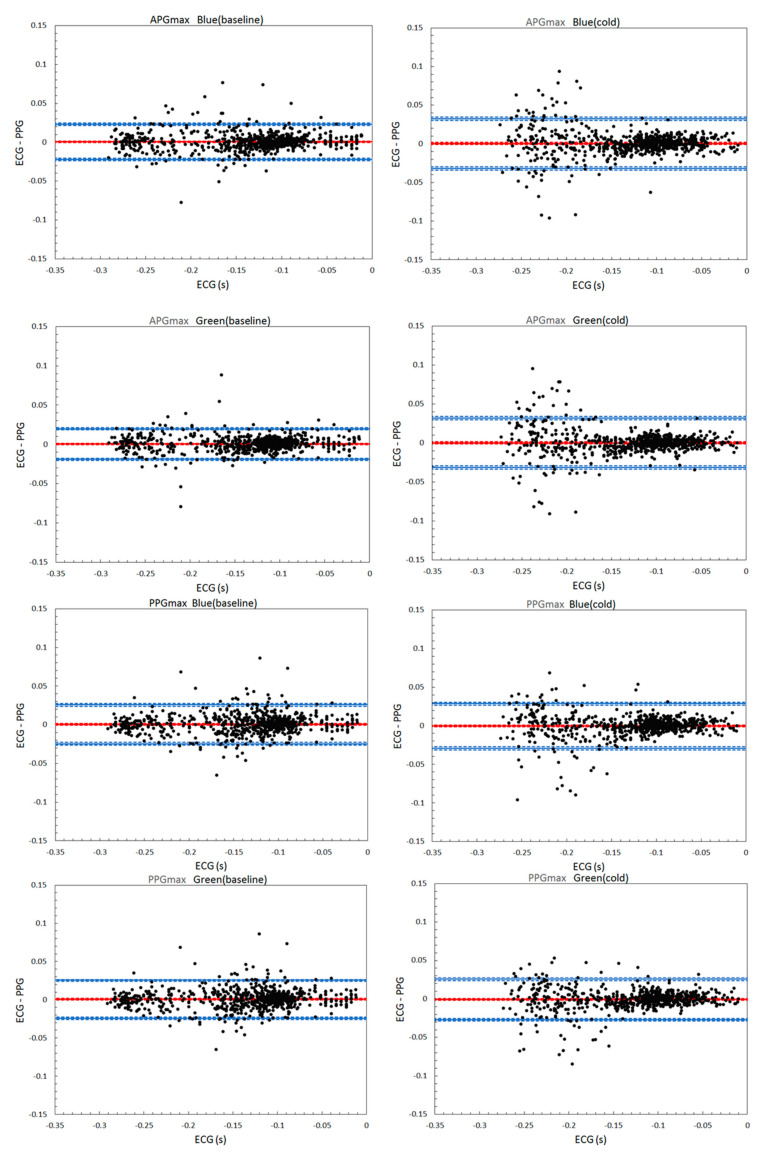

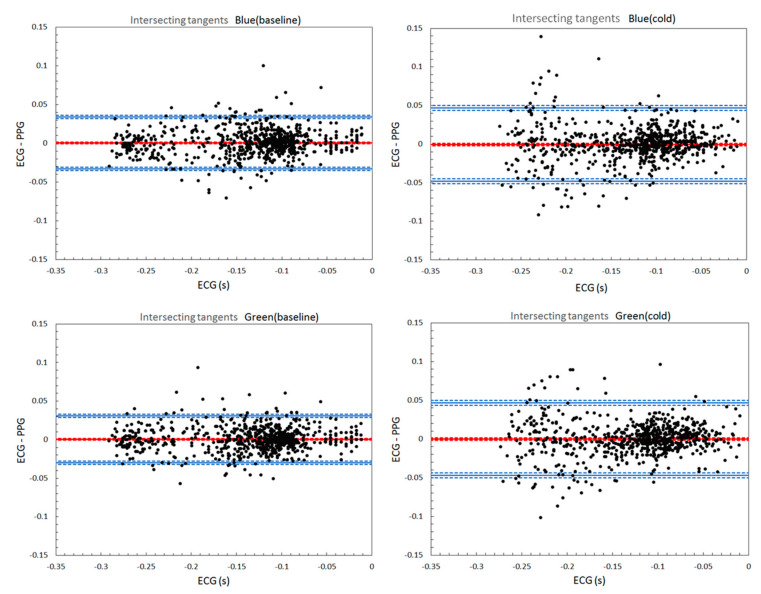
Bland-Altman plots for 720 ECG-PPG data pairs. The bias and LOA are displayed as red and blue solid lines, respectively. Their CI are displayed as dotted lines.

We also examined the differences between the characteristic points themselves. APGmin showed the largest bias values (0.0008–0.0012 s) and % bias in both wavelengths and conditions. The widest LOA and CI of bias were obtained for APGmin, and IT points. In both B and G PPG under the baseline condition, VPGmax exhibited the smallest values of bias and % bias, while in the Cold test, PPGmax demonstrated the narrowest LOA and CI of bias along with VPGmax. Thus, VPGmax outperformed other points in both wavelengths. APGmin and IT were the worst. The Bland-Altman plots are shown in Figure A1 (Appendix A). The statistical parameters, calculated for each protocol in both wavelengths, using 720 ECG-PPG data pairs from 12 subjects, are shown in Table 1.

It is apparent on the Bland-Altman plots that in the Cold test, irrespective of the wavelength and characteristic point chosen for HBIs estimation, the variabilities of the differences between methods are not consistent across the graph. The scatter around the bias line tends to increase as the ECG HBIs get shorter, especially for the intervals less than 0.67 s. The non-uniform variability can be a confounding factor in the systematic error and LOA estimates. Additionally, the Bland-Altman metrics cannot be equally valid for all measured values. To overcome this pitfall of Bland-Altman analysis, a non-parametric regression method was employed. Passing-Bablok regression analysis was chosen because it is not sensitive to outliers and sample distribution.

### 3.2. Passing-Bablok Regression Analysis

The results are presented as the intercept, slope, and residual standard deviation (RSD) with their respective 95% CI. The intercept characterizes constant (systematic) error; the slope and RSD reflect proportional and random error, respectively. If the CI of the intercept includes zero value, the absence of a constant error between the methods is assumed. Inclusion of the value “1” into the slope CI indicates the absence of significant proportional bias between the methods. A large CI for the RSD indicates a high imprecision [16].

Under both conditions, the B PPG’s regression parameters were similar to the G PPG. The systematic or proportional errors, if detected, were negligibly small. The negative sign of the constant error in both wavelengths reflects the PPG tendency to underestimate HBIs values systematically. For all points, except PPGmax, RSD is larger in the Cold test compared to the baseline. All in all, the Passing-Bablok regression results are congruent with the Bland-Altman analysis, confirming the equally good agreement of both B and G PPG with the ECG estimates for all characteristic points and conditions. The results of Passing-Bablok regression analysis are shown in Table 2.

## 4. Discussion

Theoretically, the photoplethysmographic HBIs estimates are expected to match with the intervals derived from ECG exactly. Several factors can explain a discrepancy between the measurements:

1. The influence of the pulse transit time, investigated in Kuntamalla et al.’s study [17];
2. The changes in pulse wave morphology due to the skin temperature fluctuations. On the Bland-Altman plots for both wavelengths, PPG estimates’ precision tended to degrade upon a significant drop in the skin temperature. In the cold condition, the precision reduced drastically for short HBI durations (corresponding to faster heart rates) while being less affected in longer HBIs. This finding is in line with the work of Zhang et al. [18], where the HR measurements during exercises had larger errors compared to the HR estimates during sleep and rest. Studies on PPG validation in different HBI ranges are needed to ensure proper application of the method and correct data interpretation. Probably, some cardiovascular parameters can be accurately estimated only within a certain range of HBI durations.
3. Characteristic point used for HBI estimation. Our results indicate that in both wavelengths, VPGmax algorithm yielded the best accuracy with the smallest bias, the CI of bias and LOA in the Bland-Altman test. Our findings are congruent with the earlier studies [7,9,10]. In contrast, the IT algorithm demonstrated a low level of agreement with the ECG compared to PPGmax, APGmax, and VPGmax. That is different from the previous studies [8,19]. APGmin, similarly to IT, underperformed in our study. The discrepancy in results probably originates from several factors: different skin patches being probed, properties of the light source and detector, sampling rate, signal processing methods, and so on. There are still no internationally recognized PPG research methodology standards, making it difficult to compare different approaches. For instance, in a large body of literature on photoplethysmography, the fourth-order Butterworth filter has been the most widely used filter type. Different from these studies, in our work, Chebyshev II filter was employed. Liang et al.’s work [20] showed that Chebyshev II improved the PPG signal quality more effectively than other filters (Wavelet, Butterworth, Chebyshev I, Elliptic, Median filter, Moving-average filter, FIR-hamming window, and FIR-least squares), while preserving valuable components of the signal. Maintaining the morphology of the PPG waveform is essential for characteristic point detection and for extracting additional features from the signal. The inconsistency in literature on the PPG characteristic points selection suggests that the following approaches can improve the PPG diagnostic value: (1) tailoring a characteristic point selection to signal acquisition parameters and conditions and the area of application. This approach is relevant for devices with limited computational resources; (2) using machine learning techniques.

Disregarding the experimental condition and PPG characteristic point used for HBIs calculation, the statistical tests demonstrated no significant difference between G and B PPG. Therefore, we speculate, that in some applications, they can be used interchangeably. Additional factors are favoring this assumption. Firstly, the PPG signals in our study were obtained from the palm skin. Even if the blue wavelength’s penetration depth is shallower than the green, it seems still enough to produce acceptable PPG signal quality even in the glabrous skin patch (the glabrous skin has a relatively thick nonvascularized epidermis). Therefore, we deem that the Blue PPG could give comparable or even better results on the skin with lesser epidermal thickness. Probably, the blue light’s relatively shallow penetration is compensated by its high hemoglobin absorption. Secondly, the difference in the penetration depth between the green and blue light might not be significant because both wavelengths reach only the upper part of the reticular dermis. Noteworthy, the probing depth depends not only on the light source’s wavelength but also tissues biochemistry and anatomy, the light spot size, collimated/non-collimated light, the distance between LED and skin surface, etc. [21,22]. Therefore, it is not reasonable enough to dismiss the PPG in blue light exclusively based on its probing depth.

## 5. Conclusions

We performed a comparative study on green and blue PPG signals acquired from palm skin of twelve healthy subjects at rest under two temperature conditions. The accuracy of the HBI estimates, calculated with five characteristic points (PPGmax, VPGmax, APGmax, APGmin, IT) on both PPG signal types, was evaluated for the ECG values. The interchangeability of the G and B PPG was confirmed. Thus, it can be argued that, in some healthcare applications, blue light PPG could be equally as suitable as green PPG. For example, blue light therapy is widely used in newborn jaundice, acne, and skin precancers. We speculate that combining such light-based treatments with a blue-sensitive photodetector could potentially enable cardiovascular parameters acquisition and studying the biological effects of light.

## 6. Limitations and Future Work

Several limitations that can be identified in this study.

1. The experiments were conducted only in a relatively small sample of healthy volunteers with no history of cardiovascular diseases. It is established that the PPG waveform can be influenced by many factors, such as properties of blood vessels and physical condition of an individual (sleeping hours, physical activities) [23]. For a comprehensive wavelength comparison, it is needed to recruit a more heterogeneous group of subjects.
2. The study results were based on the perfect characteristic point detection. Before HBI measurements, every characteristic point misdetected by the algorithm was manually corrected upon visual inspection. The performance of the detection algorithms in real time was not assessed.
3. B and G PPG were compared only in terms of HBIs estimation accuracy. HRV, pulse arrival time and other metrics were not calculated. Several cardiovascular parameters extracted from G an B PPG signals should be validated concerning the reference measurements in different HBIs ranges for a comprehensive evaluation.

## Figures and Tables

**Table 1:** Bland-Altman analysis results of 720 ECG-PPG data pairs from 12 subjects.

POINT	BIAS	BIAS CI Range	1/2 LOA	BAR	ALoA	BIAS %ECG
IT BB	0.0005	0.0025	0.033	0.045	0.148	−0.4
IT BC	−0.0006	0.0036	0.047	0.063	0.151	0.5
IT GB	0.0005	0.0023	0.030	0.041	0.148	−0.4
IT GC	−0.0003	0.0036	0.047	0.062	0.151	0.2
APGmax BB	0.0005	0.0017	0.023	0.031	0.148	−0.4
APGmax BC	0.0004	0.0025	0.032	0.043	0.151	−0.3
APGmax GB	0.0004	0.0015	0.019	0.026	0.148	−0.3
APGmax GC	0.0003	0.0024	0.032	0.042	0.151	−0.2
VPGmax BB	0.0003	0.0014	0.018	0.025	0.148	−0.2
VPGmax BC	−0.0004	0.0022	0.029	0.038	0.151	0.3
VPGmax GB	0.0003	0.0012	0.015	0.021	0.148	−0.2
VPGmax GC	−0.0006	0.0020	0.026	0.035	0.151	0.5
PPGmax BB	0.0006	0.0019	0.025	0.034	0.148	−0.4
PPGmax BC	−0.0004	0.0022	0.029	0.038	0.151	0.3
PPGmax GB	0.0006	0.0019	0.025	0.034	0.148	−0.4
PPGmax GC	−0.0006	0.0020	0.026	0.035	0.151	0.5
APGmin BB	0.0009	0.0026	0.034	0.046	0.147	−0.7
APGmin BC	0.0012	0.0032	0.042	0.055	0.151	−0.9
APGmin GB	0.0011	0.0023	0.030	0.041	0.147	−0.8
APGmin GC *	0.0008	0.0031	0.041	0.055	0.151	−0.6

* BB—Blue Baseline; GB—Green Cold; BC—Blue Baseline; GC—Green Cold.

**Table 2:** Results of Passing-Bablok regression analysis of 720 ECG-PPG data pairs from 12 subjects.

Characteristic Point	Intercept (95% CI)	Slope (95% CI)	Residual SD
BASELINE CONDITION			
IT Blue	−0.011 (−0.025–0.000)	1.014 (1.000–1.033)	0.020
IT Green	−0.014 (−0.027–0.000)	1.019 (1.000–1.038)	0.018
APGmax Blue	0 (0.000–0.009)	1 (0.988–1.000)	0.013
APGmax Green	0 (0.000–0.007)	1 (0.989–1.000)	0.011
VPGmax Blue	0 (0.000–0.007)	1 (0.990–1.000)	0.011
VPGmax Green	0 (0.000–0.006)	1 (0.992–1.000)	0.009
PPGmax Blue	−0.002 (−0.008–0.005)	1 (0.992–1.011)	0.015
PPGmax Green	0 (−0.011–0.000)	1 (1.000–1.014)	0.014
APGmin Blue	0 (−0.012–0.002)	1 (0.998–1.016)	0.019
APGmin Green	0 (−0.012–0.000)	1 (1.000–1.015)	0.017
**COLD CONDITION**			
IT Blue	−0.013 (−0.032–0.002)	1.019 (1.000–1.042)	0.027
IT Green	−0.010 (−0.029–0.006)	1.014 (0.993–1.039)	0.026
APGmax Blue	0 (−0.014–0.008)	1 (0.989–1.018)	0.017
APGmax Green	0 (−0.014–0.004)	1 (0.994–1.017)	0.017
VPGmax Blue	0 (−0.017–0.007)	1 (0.991–1.015)	0.016
VPGmax Green	0 (−0.000–0.000)	1 (0.983–1.000)	0.015
PPGmax Blue	0 (−0.000–0.013)	1 (0.991–1.015)	0.016
PPGmax Green	0 (−0.025–0.000)	1 (0.983–1.000)	0.015
APGmin Blue	−0.013 (−0.027–0.002)	1.014 (1.000–1.033)	0.022
APGmin Green	0 (−0.015–0.009)	1 (0.988–1.018)	0.021

## Data Availability

The dataset and LabView software used in this study are openly available in [Mendeley Data] at [doi:10.17632/6m8kmdv8ys.1].
